# Laying ghosts to rest

**DOI:** 10.1007/s11098-021-01665-6

**Published:** 2021-06-06

**Authors:** Donnchadh O’Conaill

**Affiliations:** Département de Philosophie, Univeristé de Fribourg, Av. de l’Europe 20, 1700 Fribourg, Switzerland

**Keywords:** Ghosts, Consciousness, Physicalism, Conceivability, Possibility

## Abstract

One of the most widely-discussed arguments against physcialism appeals to the conceivability of zombies, being which are physically or functionally identical to humans but which have no conscious experiences. Philip Goff (Philos Phenomenol Res 81(1): 119–139, 2010; Consci Cognit 21(2): 742–746, 2012a; in Sprevak M, Kallestrup J (eds) New waves in philosophy of mind. Palgrave, 2014) has recently presented a number of different anti-physicalist arguments appealing to the conceivability of ghosts, entities whose nature is exhausted by their being conscious. If ghosts are conceivable, this would rule out a priori physicalism. If the conceivability of ghosts entails that they are metaphysically possible, then this forms the basis for arguments against a posteriori physicalism. Drawing on work on conceivability by Peter Kung (Philos Phenomenol Res 81(3):620–663, 2010, Noûs 50(1): 90–120, 2016) and my own discussion of arguments which appeal to the conceivability of zombies (O’Conaill in Mihretu P Guta (ed) Consciousness and the ontology of properties. Routledge, New York, 2019), I shall argue that ghosts are conceivable, but that what allows us to conceive of them (our ability to make certain stipulations about the scenarios we conceive) undermines the belief that conceivability is a reliable guide to possibility. While this does not undermine Goff’s argument against a priori phyiscalism, it suggests that a posteriori physicalists need not be haunted by ghosts.

## 1

The basic idea of ghosts is that they are “creatures who are conscious but whose nature is exhausted by their being conscious” (Goff, 2010, 119).^1^ That is, a ghost is phenomenally conscious (there is something it is like to be a ghost),^2^ and it has no non-phenomenal properties other than logical properties (e.g., being self-identical) or perhaps properties logically or conceptually entailed by its phenomenal properties (e.g., the property of having experiences).^3^

Goff is careful to note that ghosts do not by definition lack physical properties (2012b, 749). For instance, if phenomenal properties are identical with physical properties, then ghosts would have physical properties. However, ghosts have no non-phenomenal properties, and so will lack any physical properties which are not identical with (or logically or conceptually entailed by) phenomenal properties. Furthermore, as we shall see below, Goff himself claims that ghosts are incorporeal, and it is reasonable to read him as claiming that they lack physical parts. In what follows, therefore, I assume that ghosts are disembodied entities.^4^

Regardless of whether or not there are (or could be) such things as ghosts, the concept of a ghost is supposed to be intuitive, one which we can understand by a familiar method:

Engage in Cartesian doubt. Consider all the propositions you believe. For each proposition such that you can’t be completely certain that it’s true, suppose that it’s false, and continue to suppose that it’s false for the rest of the exercise. Continue with the exercise up to and including the time you find yourself entertaining propositions with the knowledge that they are certainly true (Goff, 2012a, 743).

If we pursue this exercise, each of us will arrive at something like the following thought:

I can doubt that I have hands, or a body or even a brain. […] I cannot doubt that I have a conscious experience as of having hands and a body. I can doubt that there are any aspects of my nature over and above my conscious states, but I cannot doubt that I exist as a conscious thing: as a thing such that there’s something that it’s like to be it (*ibid.*; see also 2010, 124).

To entertain this possibility is to conceive of a ghost.^5^ Therefore, “Any philosopher who agrees with Descartes up to and including the Cogito has a strong prima facie obligation to accept the conceivability of ghosts” (Goff, 2010, 125). If someone doubts they can conceive of a ghost,

then she ought to tell me some alternative story about what happens when she engages in Cartesian doubt. She has to tell me whether she stops earlier or later than I do. If she agrees with me about where Cartesian doubt stops, then she must tell me why, when she reaches that point, she thinks she is not conceiving of herself as a lonely ghost. My story about Cartesian doubt is very intuitive; my opponent has to try to make some alternative intuitive (Goff, 2012a, 743).^6^

If ghosts are conceivable, this would have significant results for the philosophy of mind. First, it would rule out a priori physicalism, the view that, roughly speaking, all phenomenal facts are a priori entailed by the physical facts (Goff, 2010, 133–135; 2012a, 744–746). Second, if it is accepted that conceivability is a reliable guide to possibility, the conceivability of ghosts would provide the basis for arguments against physicalism more generally, including a posteriori versions (Sturgeon, 2000, 102, 116–117; Goff, 2010, 135–136; Janzen, 2012, 92). Third, again assuming the link with possibility, the conceivability of ghosts provides the basis for a Cartesian argument against identifying the self with a body, and against claims that the self is necessarily embodied (Goetz & Taliaferro, 2011, 93–97; Descartes, 2013, 109).^7^

I accept that ghosts are conceivable, and that this creates a serious problem for a priori physicalism.^8^ What I shall contest is the thought that the conceivability of ghosts is a good reason to accept that they are metaphysically possible. By denying that it is, the argument from ghosts against a posteriori physcialism can be blocked.

## 2

In considering the conceivability of ghosts it is worth distinguishing two questions: whether or not they are conceivable, and if they are, how they might be conceived (or what would be involved in conceiving of them, if this is possible). I shall argue in a roundabout way for a positive answer to the first question, by beginning with the second.

To clarify how we might conceive of ghosts we need to draw three distinctions. The first of these is between negative and positive conceivability. A sentence S is *negatively conceivable* if it is not a priori that S is false. *Positive conceivability* involves considering not just S but entities which are suitably related to S:

Positive notions of conceivability require that one can form some sort of positive conception of a situation in which S is the case. One can place the varieties of positive conceivability under the broad rubric of *imagination*: to positively conceive of a situation is to imagine (in some sense) a specific configuration of objects and properties (Chalmers, 2002, 150).

The suitable relation between S and the situation is as follows: were this situation to obtain then necessarily S would be true. In this case, the situation would *verify* S (*ibid.*).^9^

Goff’s preferred kind of conceivability is negative conceivability (2017, 81),^10^ but as we have seen, in outlining how we are to conceive of ghosts he appeals to a Cartesian method of doubt. It is often thought that Descartes uses positive conceivability in articulating scenarios in which we systematically doubt what we previously took to be true (*ibid.*; Chalmers, 2002, 155). Therefore, in what follows I shall first discuss what is involved in positively conceiving of a ghost, before briefly considering how what I say relates to negatively conceiving of one.^11^

The second relevant distinction is between *perceptual* and *sympathetic imagining*: “To imagine something perceptually, we put ourselves in a conscious state resembling the state we would be in if we perceived it. To imagine something sympathetically, we put ourselves in a conscious state resembling the thing itself” (Nagel, 1974, 446 n 11). For example, if asked to imagine Napoleon at Waterloo, you could put yourself in a conscious state resembling the state one would be in if one were perceiving Napoleon (e.g., you form a mental image of a short, truculent man in an early nineteenth-century military uniform); or you could put yourself in a conscious state resembling Napoleon’s own mental state when he was at Waterloo (e.g., you imagine surveying the battlefield and cursing *en français*). I shall outline the significance of the distinction between perceptual and sympathetic imagining below.

The third distinction is between *qualitative* and *stipulative content* of acts of imagining. In perceptual imagining the qualitative content will be the perceivable features of the imagined situation. On a narrow conception of the contents of perception, these are limited to primary and secondary qualities, e.g. the shape and colour of a hat. On a broader conception, they include features such as its being a hat or its wearer being present at a battle (for defence of a broad conception of perceptual content see Siegel, 2010). In sympathetic imagining, the qualitative content includes the phenomenal character of the experiences of a subject present in the situation (e.g., what it is like to survey a certain terrain from a certain standpoint). Again, one may have a narrow or broad conception of the qualitative content of sympathetic imagining (depending, for example, on whether or not one accepts cognitive phenomenology—for discussion see Bayne & Montague, 2011).

The precise limits of qualitative content (e.g., whether to adopt a narrower or broader conception of perceptual content) are not central to my argument in this paper. What is central is the thought that many acts of imagining involve content which is neither perceivable (even on a broad conception of perceptual content) nor included in the phenomenal character of the experiences of someone imagined as being in the scenario. That is, many acts of imagining involve non-qualitative or stipulative content.^12^

To illustrate the role played by stipulative content, suppose you are asked to imagine the following scenario: Napoleon’s troops did not fight Wellington’s (and Blücher’s) at Waterloo but at a nearby village, Schwaterloo, which had a landscape exactly similar to Waterloo. Furthermore, the deployment of the troops and the course of the battle were also exactly similar to those of the actual battle. To imagine this scenario perceptually, you will once again form a mental image of a short, truculent man, and the other perceptible details of the situation, which are exactly similar to those when you imagined the battle occurring at Waterloo. (I assume that the battle’s occurring at Schwaterloo as opposed to Waterloo is not itself a perceivable feature, even on a broad conception of perceptual content.) Nevertheless, it seems clear that your imagining this scenario is different from your imagining Napoleon at Waterloo, and furthermore in imagining these scenarios you can distinguish between them. You do so by stipulation: in the second scenario you stipulate that the battle is occurring at Schwaterloo.

Stipulative content allows us to imagine a huge range of scenarios, going far beyond what is perceivable or knowable to be the case, including scenarios which we know to not be the case (for instance, that Napoleon’s troops did not fight a battle at Waterloo).^13^ Indeed, the role of stipulation seems to underwrite the following methodological assumption: using stipulative content, we can imagine any scenario which is not obviously incoherent (in the next section I say a little more about possible limitations on what we can imagine). It is by means of stipulation that we can imagine a universe containing only two qualitatively identical spheres, or a colour scientist trapped in a black-and-white room, or any of the other thought experiments with which the philosophical literature is crammed. Given the power of stipulation, it is reasonable to assume that ghosts can be conceived using the method Goff outlines.^14^

It is important to note how important stipulation is in conceiving of ghosts.^15^ In following Goff’s method we are asked to imagine a being which has various experiences but no body or brain. Goff explicitly notes that we cannot do this by trying to visualise a ghost, i.e., perceptually imagining it (2010, 124). The obvious route is to sympathetically imagine it, which involves imagining ‘from the inside’ certain experiences (as of seeing a tree, as of feeling a twinge in one’s leg, etc.). But note that the qualitative content of this imagining is not sufficient for what one imagines to count as a ghost – one must also add that the entity having these experiences has no brain or body. This extra content is stipulative, in two ways. First, it involves specifying the *absence* of certain entities or properties, i.e., in this scenario there is an entity with no body or brain.^16^ Second, it involves the claim or assumption that the *very entity* whose experiences you are imagining ‘from the inside’ lacks these properties. That is, imagining a ghost from inside involves combining perspectives (the ghost’s own perspective, i.e., what it is like for it to have its experiences, and a perspective from the outside which includes information that the ghost might not itself be able to discover, i.e., that it is a ghost). Both of these claims or assumptions (the absence of certain entities and the combining of different perspectives) are stipulative, in that they are not included in or entailed by the qualitative character of one’s imagining (for more on this see O’Conaill, 2019, 62–65). Without such stipulative content, ghosts would be inconceivable.

The suggestion that imagining ghosts requires stipulations which go beyond qualitative content might be questioned as follows.^17^ The thought would be that imagining ghosts does not require imagining absences, but imagining properties which exclude having a body, e.g., having the ability to penetrate solid objects; and, it might be suggested, this can be imagined without stipulations. For example, I imagine having an experience of walking towards a wall, then seeing what is on the other side of the wall from a point on the other side. This experience, it might be argued, rules out my being a corporeal entity without my having to stipulate this.

But simply imagining this succession of experiences would not suffice for what I have imagined to count as an incorporeal entity. First, it needs to be the case that what I imagine passing through is a solid object rather than, e.g., a simulacrum of a wall. Second, the experiences I imagine cannot be hallucinations. Both of these possibilities must be ruled out, and on the face of it this requires stipulations which go beyond the qualitative content of my imagining.

Thus far in this section I have argued that positively conceiving of ghosts depends on stipulative content. To negatively conceive of a ghost is simply to consider a proposition such as ‘A ghost exists’, and to not find it incoherent. It is plausible that this also requires stipulative content. The very concept ‘ghost’ we are using is the product of stipulation: we stipulate that it is a concept of an entity whose nature is exhausted by its phenomenal characteristics, which is disembodied, etc. It is arguable that every complex concept is the product of such stipulation, which allows us to join together other concepts; but even if this is not true of all complex concepts, it is extremely plausible for the concept of a ghost.

## 3

Stipulative content allows us to imagine far-fetched scenarios, but this very flexibility threatens to undermine the epistemic link between conceivability and possibility. It is plausible that we can conceive of scenarios which are metaphysically impossible.^18^ For instance, it is widely thought that it is impossible that I could have had different biological parents, but it seems I can imagine this: e.g., I visualise a scenario of an infant and a couple, and I stipulate that the infant is me and that this couple are my biological parents. Or I can imagine an American teenager travelling back in time and altering the past; again, it is widely thought that this is impossible, but it seems that I can understand a story where this is recounted (*Back to the Future*), and it is plausible that to understand a story is to conceive of the events it describes as occurring.

Why would stipulation allow us to conceive of metaphysically impossible scenarios? One answer is that stipulation is very close to thinking, and it seems plausible that we can think about metaphysically impossible scenarios. More precisely, stipulation in itself is relatively unconstrained, and those constraints which do apply seem to be of little epistemic value. Kung suggests that the principle constraint on stipulations is that we not be absolutely certain that they are impossible: “so long as we find P believable, epistemically possible in the strongest sense that it is true for all we know for certain, or possibly true for all we know for certain, we will be able to imagine P via stipulation” (2010, 634). As Kung points out, one’s lacking certainty that P is impossible does not seem relevant to the issue of whether P is (metaphysically) impossible (*ibid.*). Kung also considers other possible constraints on stipulation (*op. cit.*, 635–636) and argues, in my opinion convincingly, that these have little epistemic force.

Note that this conclusion goes beyond the claim that imagining by stipulation sometimes fails as a guide to possibility; that is, that we occasionally imagine impossible scenarios. As Stephen Yablo points out, this claim would not by itself count against imagination being a guide to what is possible (1993, 13). The point I am making, following Kung, is not that imagination by stipulation is not an *infallible* guide to possibility, but that it is not a *reliable* guide. It is not reliable because stipulation in itself is not constrained in a way which is epistemically relevant, i.e., a way which establishes a close relation between what we stipulate and what is metaphysically possible.

This argument stands in need of further development: in particular, the precise notion of reliability to which I am appealing would need to be clarified (e.g., how weak would the relation between what we can stipulate and what is possible have to be before stipulative imagination was no longer a reliable guide to possibility?). But the intuitive idea is clear enough. Compare with testimony. A person’s testimony can be a reliable guide to certain things, even if she occasionally makes mistakes. However, the thought that her testimony is reliable assumes a fairly close relation between what she is inclined to report and what is actually the case. If she is not constrained in a way which makes her inclined to tell the truth (at least as she sees it), then what she reports will not be a reliable guide to what is the case (or, at the very least, we would have no reason to take what she says as a reliable guide). This conclusion is reasonable even if we cannot state exactly how often her testimony would have to be mistaken for it to be unreliable.^19^

As noted in Sect. 2, imagining ghosts requires different kinds of stipulation. First, one must stipulate the absence of certain properties from the imagined scenario, or at least from certain entities in this scenario. Second, one must combine one’s imagining from inside with external information about the scenario (namely, that the entity having certain experiences is an entity which lacks certain properties). Without these stipulations, one will not have succeeded in imagining a scenario containing a ghost (the scenario one imagines might contain a ghost, but it might simply be one containing ordinary human subjects). As we have seen, stipulation by itself is not epistemically constrained in any significant way. So the stipulation cannot itself be relied upon to establish the possibility of the imagined scenario.

Of course, the act of imagining this scenario also contains non-stipulative content, e.g., the qualitative content of imagining what it would be like to have certain experiences. But this content on its own is not sufficient to establish that what one is imagining is in fact a ghost; it is compatible with what you are imagining turning out to be a flesh-and-blood human. And, crucially, there is little reason to think that this qualitative content can constrain the stipulative content in an epistemically relevant way. That is, there is little reason to think that it systematically rules out stipulations which allow us to imagine impossible scenarios.^20^ Without a way of ruling out such stipulations, we have no assurance that our ability to conceive of ghosts provides reliable evidence that they are metaphysically possible.

## 4

The proponent of ghost arguments against physicalism has a number of possible responses to the criticism just outlined. She could give up the claim that conceivability provides a reliable guide to possibility, and thus give up the ghost argument against a posteriori physicalism, while continuing to insist that the conceivability of ghosts provides a powerful argument against a priori physicalism. Or she could challenge the account of positive conceiving or imagining outlined in Sect. 2. I shall not address this suggestion here except to refer the reader to previous work articulating and defending this account (e.g., Kung, 2010, 2016; O’Conaill, 2019).

A third strategy would be to accept the account of conceiving outlined above but to argue that stipulative content can be constrained in epistemically relevant ways. That is, even if conceiving by itself is not a reliable guide to what is possible, there are further constraints which can be placed upon how we conceive; and, the suggestion is, conceivability constrained in these ways is a reliable guide to possibility. In the remainder of this paper I shall consider two ways which have been outlined by Goff for constraining what we conceive in order to make it a reliable guide to what is possible.

The first of these ways appeals to what Goff terms the *Cartesian Principle* (CP): “In so far as a conception is of phenomenal properties qua phenomenal properties—that is, in terms of what it is like to have them—there can be no gap between conceivability and possibility” (2010, 125). That is, suppose I try to conceive of a scenario ‘from the inside’, and suppose my conceiving includes no content other than which phenomenal properties are instantiated (i.e., what it is like for the subject whose perspective I imaginatively assume). The thought is that if I follow this procedure and if this scenario is conceivable, then it is possible.

In what follows I shall assume CP is correct.^21^ Goff develops his argument as follows:

If CP is true, if there cannot be a gap between conceivability and possibility regarding a conception of phenomenal properties (qua phenomenal properties), then it is difficult to see how a gap between conceivability and possibility could open up when one goes from conceiving only of phenomenal properties to conceiving of a thing which exemplifies only phenomenal properties. To apply this to the case in question, it is difficult to see how a gap between conceivability and possibility could open up when one moves from conceiving of phenomenal properties to conceiving of a ghost, i.e. a creature which has only phenomenal properties (2010, 127).

The obvious worry with this argument is that it involves a change in scope, from conceiving of a scenario *only with respect to P* (i.e., conceiving of a scenario only with respect to the instantiation of phenomenal properties qua phenomenal properties) to conceiving of a scenario in which *only P is instantiated*, i.e., conceiving of a scenario in which these phenomenal properties are the only properties instantiated. In the first case, one in effect conceives of a scenario and ignores all its features other than P. In the second case, one conceives of a scenario in which P is the only feature (apart from merely logical properties, or properties logically or conceptually entailed by instantiations of P).

The significant of this difference can be brought out as follows. In the first case, one can conceive of a scenario solely by using phenomenal concepts (e.g., the concept ‘P’). But one cannot conceive of a scenario in which P is the only property instantiated by using only the concept ‘P’ (or even by using only the concept ‘P’ and a concept for whatever it is which instantiates P). The content of one’s conceiving must include stipulations about the rest of the scenario, e.g., that the bearer of P has no other properties.^22^ So while the scenario imagined in the second case might contain fewer entities or types of entities than that imagined in the first case, the act of imagining in the second case requires using more concepts.

So even if we accept CP with regard to one’s concept ‘P’, it does not follow that when one conceives of a scenario in which P is the only property, there is no gap between this conceiving and the possibility of this scenario. The extra stipulations required (e.g., that there are no other properties) involve content which goes beyond my concept of ‘P’. And it cannot be assumed without argument that this extra content does not open a gap between conceivability and possibility.

## 5

The second line of thought appeals to the notion of transparent concepts: “A concept is *transparent* just in case it reveals the nature of the entity it refers to, in the sense that it is a priori (for someone possessing the concept and in virtue of possessing the concept) *what it is for that entity to be part of reality*” (Goff, 2017, 15).^23^ Goff gives as an example of a transparent concept the concept ‘sphericity’: the thought is that to grasp this concept is to understand the essence or nature of the property sphericity, i.e., what it is for something to be spherical (it is for that thing to have a centre which is equidistant from all points on its surface). In contrast, a concept is *opaque* if it does not reveal anything (or at any rate, very little) about the essence of the entity or entities which satisfy it. For instance, the concept of water is opaque. The nature of water is, we may assume, a matter of a substance having a certain chemical composition, but this composition is not revealed by the concept itself.

The notion of the nature or essence of an entity (Goff uses these terms interchangeably) here is *non-modal*, in the following sense: an entity’s essence need not include all of its necessary features (non-modal essence is famously defended in Fine, 1994). Put another way, we can distinguish between *what it is* for an entity to be part of reality (its essence or nature) and *what is necessary* for an entity to be part of reality, which includes its essence and also necessary conditions for that entity to exist which do not belong to its essence. A transparent concept of an entity reveals a priori its essence, but it does not follow that it reveals all of what is necessary for that entity to exist. For instance, Goff thinks that phenomenal concepts are transparent, in that the essence of the phenomenal properties which satisfy them is revealed when we attend to instances of these properties, i.e., particular experiences. However, he thinks there are limits to what can be revealed about phenomenal properties in this way: “Pain is necessarily such that 100 × 127 = 12,700, but it is not plausible that this is revealed to me when I attend to a throbbing in my shin” (Goff, 2015, 124). I shall return to the distinction between essence and necessary conditions presently.

A *transparent thought* involves no singular concepts, and each of the property- or kind-denoting concepts it involves are transparent (Goff & Papineau, 2014, 753; Goff, 2017, 99).^24^ In thinking such a thought “one understands (or has a priori access to) what it would be for the possible fact being conceived of to obtain” (Goff, 2017, 97). The *Transparency Conceivability Principle* (TCP) is the claim that if a transparent thought is conceivably true, then it is possibly true (*op. cit.*, 100; see also 2010, 136).^25^ As an example of this claim, if we have a transparent conception of pain and of c-fibres firing then we “can infer from the conceivability of their separation, to the genuine possibility of their separation” (Goff & Papineau, 2014, 755–756).

TCP suggests a way in which we can explain the conceivability of metaphysically impossible scenarios. If one conceives of a scenario using opaque concepts, this leaves open that what one conceives of is impossible. For instance, if one’s concept of ‘negative charge’ is ‘whatever my physicist friend is researching’, the nature of negative charge is not revealed to me and I am free to coherently conceive of, e.g., electrons as not having negative charge (Goff, 2014, 9; Goff & Papineau, 754). A further claim would be that all instances of conceiving the impossible can be explained in terms of opaque concepts. As Goff puts it, “We can plausibly explain” gaps between conceivability and possibility “in terms of the opacity of thought […] it is plausibly our ignorance of the nature of what we conceiving of that opens up the Kripkean gap between conceivability and possibility” (2017, 9). Indeed, he suggests that “the metaphysically possible worlds are those which can be conceived of under a transparent conception” (Goff & Papineau, 2014, 753).

It certainly seems correct that if one does not know the nature of what one is conceiving, one may thereby be more prone to certain kinds of error, and so transparent conceiving may be more reliable as a way of discerning metaphysical possibility than non-transparent conceiving. But this does not establish that transparent conceiving is reliable enough to count as a source of modal knowledge, let alone Goff’s much stronger claim that what is metaphysically possible just is what is transparently conceivable. To properly assess this issue, we need to consider the following question: if one conceives of a scenario using only fully transparent concepts, does this entail that this scenario is metaphysically possible, or that one thereby has good grounds to take this scenario to be possible?

The notion of a transparent thought is couched in terms of transparent concepts. Goff claims that in thinking such a thought, “one understands (or has a priori access to) what it would be for the possible fact being conceived of to obtain” (2017, 97). This is correct, but there is a crucial distinction between *what it would be for a fact F to obtain* and *what is necessary for a fact F to obtain*. This follows from the general distinction outlined above between what it is for an entity *x* to be part of reality and what is necessary for *x* to be part of reality. Likewise, the essence of a property, what it is for that property to be instantiated, does not include all of the conditions which are necessary for it to be instantiated.

Once these distinctions are made clear, it should be obvious that we cannot conclude from one’s having a transparent concept of some fact or some property that one thereby understands all of the conditions which are necessary for that fact to obtain or that property to be instantiated. That is, grasping the essence of some property P does not in itself rule out the possibility that there may be some other conditions which must hold for it to be instantiated, conditions which one may not grasp even if one grasps the entire essence of P.^26^ Suppose one such condition is that P cannot be instantiated unless some other property, Q, is instantiated. It seems that one could not rule out there being such a condition even if one were to grasp the full essence of P and of Q (i.e., there might be some necessary connection holding between P and Q which is included in neither the full essence of P, nor that of Q, nor of both). That is, the transparent concepts of P and Q would not, on this interpretation, rule out the possibility of a necessary (non-essential) connection holding between them, such that each instantiation of P would necessitate an instantiation of Q. Therefore, the thought ‘P and not-Q’ might be transparent and yet it might be metaphysically impossible for P to be instantiated without Q. Therefore, TCP is false: if one conceives of a scenario using only transparent concepts, it does not follow that this scenario is metaphysically possible.^27^

## 6

Goff offers two lines of argument in defence of TCP. First, he suggests that a counterexample to TCP would be a *strong impossibility*,

a thought T that cannot possibly be true, despite the fact that (A) a complete understanding of the essence of T’s truthmaker (i.e., the fact that would have to obtain for T to be true) in conjunction with (B) idealized rational capacities, would not allow one to work out that T cannot possibly be true (2017, 123).

He suggests that it is intuitively wrong to think that “coherence and possibility are so radically divorced from each other” (*ibid.*).

But it is not clear why it would be wrong to divorce coherent transparent conceiving from *metaphysical* possibility. Transparent conceiving plausibly corresponds exactly with what we might term *essential possibility*, that is, what is possible given the essences of all entities. But the question is whether all essential possibilities are metaphysically possible. On the “standard model” of modalities, the metaphysical possibilities are a sub-group of the logical possibilities (Vaidya, 2015). The suggestion under consideration now is that the metaphysical possibilities are also a sub-group of the essential possibilities. The explanation for this would be that what is metaphysically possible is constrained both by the essences of all entities and by metaphysical principles which are not grounded in the essence of any entity or entities.^28^

Of course, I have not provided any independent reason to think that there are such metaphysical principles. But in this debate the burden of proof lies with the proponent of ghost arguments against a posteriori physicalism, to provide reasons to think that there are no such principles. She is putting forward an argument against physicalism, and to make this argument work she must justify the move from the conceivability of ghosts to their being metaphysically possible. As long as metaphysical principles which are not essentially grounded cannot be ruled out, then we cannot move from what is transparently conceivable to what is metaphysically possible, even if what is transparently conceivable is essentially possible.

Another way Goff defends TCP is by appealing to the programme of *essentialism*, which aims to explain modal truths in terms of truths concerning essences (Fine, 1994; Lowe, 2012). Goff regards this as the most promising programme to account for modal truths (2017, 123–124). It is true that essentialism has flourished recently, but it has also attracted a flourishing opposition (e.g., Noonan, 2018; Romero, 2019; Teitel, 2019; Whittle, 2010; Wildman, 2021). So this defence of TCP is best understood as conditional: if one has reason to think that essentialism is a plausible account of modal truths, then one will have reason to accept TCP. I take it that the antecedent of this conditional, while certainly not obviously incorrect, is at the very least open to question. And again, given the dialectic the onus would be on the proponent of ghost arguments to provide reasons to think that the objections to essentialism can be overcome.

A final reply to my criticism of TCP would be as follows: if we cannot rule out strong impossibilities we cannot take transparent conceivability to be an infallible guide to what is possible, but surely the fact that we cannot rule out strong impossibilities is not sufficient to show that transparent conceivability is not reliable? It is widely agreed that a source of warrant or justification can be reliable while being fallible. The worry is that at best my argument establishes that transparent conceivability is fallible (or, to be more precise, that we have reason to not take it to be infallible); but this falls short of establishing that it is not reliable. Furthermore, it might be added, I have not produced any counterexample to TCP.

In response to this objection, again the burden of proof surely lies with proponents of TCP to give reasons to think that transparent conceiving *is* a reliable guide to possibility, rather than on their opponent to show that it is *not*. To develop this response in more detail, the general worry about stipulative content outlined in Sect. 3 can be applied to transparent conceiving. In transparently conceiving a scenario with P and not-Q, stipulation is still required (assuming that the transparent concepts of P or Q do not themselves rule out Q’s being instantiated along with P, or Q needing to be instantiated in order for P to be instantiated). To the extent that the general arguments against the reliability of stipulation work, they would also seem to apply here, at least prima facie. Furthermore, the fact that I have not provided counterexamples to these specific stipulations does not by itself entitle us to suppose that these stipulations are reliable. Again, comparison with testimony is useful here—if we have reason to think that John’s testimony is unreliable because what he is inclined to report is not, in general, closely tied to the truth, one does not need to be able to produce a specific example of him getting it wrong with regard to a particular subject-matter in order to doubt whether his testimony with regard to that subject-matter is reliable.

## 7

Arguments appealing to the conceivability of ghosts develop a long-standing philosophical tradition reaching back at least to Descartes. They directly threaten a priori versions of physicalism, and if conceivability is a reliable guide to possibility they would threaten a posteriori versions as well. I have argued that what allows us to conceive of such exotic scenarios as ghosts (the use of stipulation in imagining) undermines the thought that conceivability is a reliable guide to possibility; because stipulations are not constrained in epistemically relevant ways, we have good reason to think that we can imagine metaphysically impossible scenarios. I have further argued that two proposals to constrain how we imagine different scenarios (appealing to the Cartesian Principle and to transparent thoughts, respectively) do not succeed; we do not have good reason to think that even constrained in either of these ways, imagining would be a reliable guide to possibility. This is not to suggest that no such constraint could be developed, but until this happens proponents of a posteriori physicalism need not fear ghosts.

## Footnotes

1 Arguments based on conceiving of such beings have a longer history than those based on conceiving of zombies. Descartes offers one such argument in the Sixth Meditation (2013, 109). For more recent examples see Goetz & Taliaferro (2011), Swinburne (2018). A similar argument is discussed critically in Sturgeon (2000).

2 In what follows ‘possible’ and its cognates refer to metaphysical possibility unless otherwise stated.

3 I say ‘logically or conceptually entailed’ rather than ‘metaphysically necessitated’ because, as shall become clear, one of the major issues raised by the idea of a ghost is whether instantiations of phenomenal properties metaphysically necessitate instantiations of non-phenomenal properties (other than properties such as the property of having experiences).

4 In different papers (2010, 2012a, b, 2014) Goff describes different versions of ghosts. These descriptions contain details which go beyond what I have outlined in the main text. In what follows I shall focus on the basic characterisation offered above.

5 A complicating factor here is that Goff accepts that even if ghosts are possible, it may be that it would not be possible for *him* to exist as a ghost (2010, 128; see also Zimmerman 2003, 19–20). This may create problems for arguments for the possibility of ghosts which rely on first-personal methods of conceiving of ghosts, but I shall put these aside in what follows.

6 Goff suggests that this method of Cartesian doubt means that the conceivability of ghosts is not vulnerable to some objections facing the conceivability of zombies (2010, 134–135). For doubts on this score see Diaz-Leon (2012), Goff replies in his 2012b.

7 Goff outlines a similar argument (2014, 8–9) but ultimately rejects it. He also argues that the conceivability of ghosts threatens another position which he terms ‘funny physcialism’ (2010, 129–133). This is a position on which the physical nature of entities includes categorical properties which do not show up in the way such entities are characterised in physics.

8 This is a significant concession to the importance of the conceivability of ghosts. That said, most contemporary physicalists think physicalism must be justified on a posteriori grounds, and this concession does not by itself undermine that position.

9 See also Sturgeon’s discussion of experiential imagination as the primary (though not the only) source of conceivability (2000, 107–109).

10 Strictly speaking, it is ideal negative conceivabilty. In what follows I shall assume that all conceivability under discussion is ideal, i.e., the conceiver is fully rational and makes no inferential mistakes.

11 It is worth noting that the term ‘imagination’ as I shall use it differs from Descartes’s use. Descartes assumes that “imagining is simply contemplating the shape or image of a corporeal thing” (2013, 39). I follow Goff in using the term ‘imagining’ in a broader sense, not tied so closely to sense perception (2017, 81 n6). In this broad sense, it is possible to imagine scenarios containing incorporeal entities.

12 The distinction I shall draw between stipulative and qualitative content follows that drawn by Kung (2010, 623–625). The basic idea of distinguishing qualitative and non-qualitative content is found in numerous other places in the literature, e.g., Chalmers (2002, 150), Berto & Schoonen (2018, 2707–2708).

13 In some cases there may be doubts as to whether we can actually imagine these scenarios, but I think that the best way to fit with our standard practice is to assume that we do imagine them unless we have specific reason to think that, e.g., the scenario in question is logically incoherent. For a general defence of this approach see Kung (2016).

14 The conceivability of ghosts is challenged by Greg Janzen, who claims that “to conceive of oneself as a conscious creature of any kind—i.e. as a creature who has visual, tactile, auditory, cognitive, etc. experiences—is, ipso facto, to conceive of oneself as an embodied creature” (2012, 93–94). The reason for this is that conscious states are essentially perspectival: “a subject has a conscious experience only if she has the experience from her present perspective” (*op. cit.*, 94). When I see something, I see it from where I am located, and even if I am hallucinating “there is still a perspective from which I undergo my hallucinatory experience: I undergo my experience from here, from my present perspective” (*ibid.*). Therefore, one can experience (or think) only if one is spatially located: “One can consciously think about Fitch’s paradox, for example, only if one thinks about it from one’s present position in space” (*ibid.*). I shall not be able to properly address this line of thought, but it seems in danger of conflating two distinct claims: (a) experiences are ‘spatially structured’, in terms of their contents or modes, i.e., one experiences things *as from* a point in space; (b) the experiencer must actually *be* spatially located. The examples of perceptually perspectival experiences support claim (a), but they do not clearly support claim (b); but claim (b) is what Janzen needs for his argument to work. It is also worth mentioning that even if it is impossible for disembodied beings to have experiences, it does not follow that one cannot conceive of such beings.

15 Stipulation is also crucial in conceiving of ghosts as impossible, i.e., in conceiving of subjects as identical with their bodies (see Zimmerman, 2003).

16 It is important to note the difference between stipulating that certain entities are absent from a scenario and merely failing to imagine their being present. The latter leaves open whether or not the scenario includes these entities, whereas it is crucial to the imagined scenario’s containing ghosts that these entities are absent. These entities could also be ruled out by listing all the entities in the scenario and adding a ‘that’s-all clause’ (Goff, 2012b, 750). But this is itself a stipulation, that the scenario contains nothing except what has been listed.

17 Thanks to Olivier Massin for formulating this objection.

18 These examples are each adopted from work by Kung (e.g., 2010, 626); for further defence of the claim that we can imagine impossible scenarios see Ichikawa & Jarvis (2012), Berto & Schoonen (2018). There are probably no clearcut examples of scenarios which are both conceivable and metaphysically impossible. The examples I shall offer are best understood conditionally, i.e., if one accepts that the scenario described is metaphysically impossible, then they can serve as examples of impossible but conceivable scenarios (see Byrne, 2007, 129–130).

19 A referee pointed out that the order of explanation is different in the cases of imagining and testimony, respectively. In the case of imagining, we move from the lack of constraint to the conclusion that imagining is not a reliable guide to what is possible. In the case of testimony, we typically move from discovering that the person has been getting things wrong to the conclusion that what the person is inclined to report is not constrained by what is the case. Nevertheless, the analogy works despite this difference. This is because the point of the analogy is a limited one: in each case, we are entitled to conclude that an information source is not reliable enough to be trusted, even if we cannot say exactly how weak the relation is between what the source tells us and what is the case (or what is possible). This can be true even though the way we reach this conclusion is different in the cases of imagining and testimony.

20 The qualitative content might allow us to rule out certain stipulations, e.g., that no experiences are occurring in this situation. But it cannot rule out the stipulations which are relevant to conceiving of ghosts.

21 CP is in effect the claim that phenomenal concepts are *transparent* (see Sect. 5).

22 A related point is made by Dean Zimmerman (2003, 24–25).

23 I assume, following Goff, that what it is for a property to be part of reality is for it to be instantiated. Likewise, I assume that what it is for a fact to be part of reality is for it to obtain, and I assume that for a fact to obtain is for a property or properties to be instantiated or for two or more entities to stand in some specific relation. The argument I shall outline in this section does not depend upon these assumptions, but they simplify its presentation.

24 Goff adjusts his conception of transparency in his 2021, but in ways which are not relevant to the points I shall make.

25 This is adapted slightly from Goff’s own formulation in terms of transparent sentences.

26 One example of this is Goff’s own suggestion that pain is necessarily such that 100 × 127 = 12,700. Another example concerns the existence of any individual concrete object. Given standard set-theoretic assumptions, any individual concrete object is necessarily a member of its own singleton set, but on a non-modal conception of essence it is not essential to any individual concrete object that it be a member of such a set. It seems possible for someone to have a transparent concept of the fact that some individual concrete object exists without understanding anything about singleton sets (this person may be ignorant of set theory). Therefore, it seems possible for someone to have a transparent concept of the fact that some individual concrete object exists and not to thereby understand a condition which is necessary in order for this fact to obtain.

27 Note that this objection is different to the thought that our conception of an entity might include some but not all of its essence, leading us to mistakenly think that whatever is not included in our conception is not part of its essence. In Goff’s terminology, this worry would be that our concepts may be *translucent* rather than transparent (2017, 102). In contrast, the objection in the main text applies even if it is accepted that the concepts are fully transparent—it relies on the distinction between essential truths and necessary truths about an entity, rather than on the distinction between a full and partial understanding of that entity’s essence.

28 A similar point is made by Joseph Almog, who points out that a thought might not be possibly true even if it is consistent with the essence of the entities it is about (2002, 36–37).
